# Colloidal Dispersion of a Perfluorosulfonated Ionomer in Water–Methanol Mixtures

**DOI:** 10.3390/polym10010072

**Published:** 2018-01-14

**Authors:** Sinan Li, Ken Terao, Takahiro Sato

**Affiliations:** Department of Macromolecular Science, Osaka University, Toyonaka Osaka 560-0043, Japan; crashlis@yahoo.co.jp (S.L.); kterao@chem.sci.osaka-u.ac.jp (K.T.)

**Keywords:** Nafion, ionomer, colloidal dispersion, small-angle X-ray scattering, light scattering, electrostatic interaction, hydrophobic interaction

## Abstract

We have investigated the dispersion state of a perfluorosulfonated ionomer (PFSI; Nafion^®^) in aqueous dispersion and the effect of methanol (MeOH) added to the aqueous dispersion by small-angle X-ray scattering (SAXS) as well as static and dynamic light scattering (SLS and DLS, respectively). Although both electrostatic and hydrophobic interactions of PFSI are expected to be strong in the dispersions, SAXS profiles obtained were satisfactorily fitted by the spherical particle model of a bimodal molar mass distribution. The rod-like aggregate model proposed in previous papers was denied at least for the present PFSI dispersion. Although the SAXS profiles exhibited a weak peak and the auto-correlation functions of DLS showed a log-time decay by the “repulsive cage effect” due to the long-ranged electrostatic interaction among PFSI particles, the concentration dependence of SLS results was probably normal because the cancellation of the electrostatic and hydrophobic interactions. The addition of MeOH into the aqueous dispersion of PFSI weakened both the hydrophobic and electcrostatic interactions of PFSI, and it is rather difficult to classify whether MeOH is a good or poor solvent (dispersant) for PFSI.

## 1. Introduction

Ionomers are defined as polymers consisting of a hydrophobic backbone and ionic side chains of a relatively low-content (up to circa 10 to 15 mole %) [1]. Therefore, ionomers can be regarded as a kind of amphiphilic polymer. However, they are usually insoluble and do not form micelles in water. Ionomers have unique mechanical and electric bulk properties and are used as semipermeable membranes, thermoplastic elastomers, and so on.

**Scheme 1:**
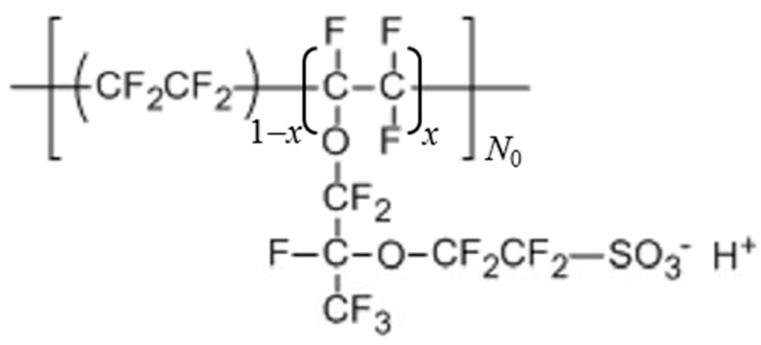
Chemical structure of perfluorosulfonated ionomer (PFSI) used in this study.

A perfluorosulfonated ionomer (PFSI), Nafion^®^, is an ionomer possessing a perfluorinated backbone and a small content of sulfonated side chains (see Scheme 1). PFSI was developed by Du Pont in the late 1960s and is now used as the proton exchange membrane for fuel cells. PFSI is commercialized as the aqueous dispersion, and its membrane is prepared by dispersion casting. Therefore, its membrane properties may be strongly dependent on the dispersion state of PFSI before casting. So far, many studies were carried out on the dispersion state of PFSI in aqueous dispersion as well as in polar dispersants [2,3,4,5,6,7,8,9,10,11,12,13]. However, the very strong opposite interactions and the electrostatic repulsion and hydrophobic attraction among PFSI particles in the dispersion make the characterization of the PFSI dispersion difficult.

In 1986, Aldebert et al. [3] reported that small-angle neutron scattering (SANS) from aqueous PFSI dispersions provided scattering functions with a broad peak. From the peak position, they estimated the average inter-particle distance, which showed a reciprocal square root dependence on the PFSI concentration. From this result, the original authors concluded that PFSI exists in an aqueous medium as a rod-like particle. Afterward, several research works supported or assumed this conclusion of the rod-like particle. However, the scattering function is the product of the particle scattering function and structure factor, so that its peak position does not necessarily provide the information of the average inter-particle distance in the colloidal solution. As shown in Appendix A, the theoretical scattering function for the hard-sphere solution shows a peak at high sphere concentration, but the reciprocal of the peak position does not obey the reciprocal cubic root dependence on the concentration expected for the inter-particle distance of neighboring spheres. The broad peak of the scattering function is affected by both the particle scattering function and structure factor, so that the peak position is not directly related to the inter-particle distance of neighboring spheres.

In the present study, we have investigated the dispersion state of a commercialized PFSI in water and water–methanol (MeOH) mixtures with different MeOH contents by static and dynamic light scattering (SLS and DLS) as well as small-angle X-ray scattering (SAXS). To avoid additional aggregation of PFSI particles, we did not add salt in the solutions. Therefore, there is the strong long-ranged electrostatic interaction among PFSI particles in the solution, but we can also expect the strong hydrophobic interaction among perfluorinated backbone chains, which may be comparable to the electrostatic interaction when two particles come close to each other [14]. Therefore, the characterization of the PFSI dispersion is challenging work.

## 2. Materials and Methods

### 2.1. Sample and Solutions

A commercialized perfluorosulfonated ionomer (PFSI) sample, Nafion^®^-DE1021 (Aldrich Chemical Co., St. Louis, MO, USA), was used in this study. This sample was supplied in the aqueous dispersion state and as the acid form. The concentration of the original aqueous solution was 10 wt % of polymer.

The molar mass per sulfonate group (or the equivalent weight, *EW*) for Nafion^®^-DE1021 was reported to be 1100. From this *EW*, the mole fraction of the charged monomer unit *x* was calculated to be 0.13, and the average monomer unit molar mass ${\bar{M}}_{0}$ to be 146 g/mol. Because of no suitable solvent for PFSI, the degree of polymerization *N*_0_ was not determined for the present PFSI sample.

The original aqueous PFSI dispersion was directly diluted with pure water in order to prepare test solutions of different polymer mass concentrations *c*. The original solution was also diluted by mixtures of water and methanol (MeOH) to prepare the test solutions with water weight fractions *w*_H_2_O_ from 0.2 to 0.8. To avoid secondary aggregation of PFSI, no salt was added to the solutions. All the test solutions were stirred overnight at room temperature during the dissolved process. All the test solutions were filtrated with a 0.5 μm pore-size membrane filter before static light scattering (SLS) and small-angle X-ray scattering (SAXS) measurements.

### 2.2. SLS and DLS Measurements

SLS and DLS measurements were performed using an ALV/SLS/DLS/5000 light scattering instrument equipped with an ALV-5000 multiple digital correlator (ALV-GmbH, Langen, Germany). All measurements were carried out at 25 °C. Vertically polarized light with the wavelength 532 nm emitted from an Nd:YAG laser was used as incident light. The light scattering system was calibrated using toluene as the reference material to determine the absolute excess Rayleigh ratio *R*_θ_.

The weight average molar mass *M*_w_ and the second virial coefficient *A*_2_ were determined using the Guinier plot

(1) $$\ln\left(\frac{R_{0}}{Kc}\right)=\ln M_{w}-2A_{2}M_{w}c$$

where *R*_0_ is the excess Rayleigh ratio at the zero scattering angle, *c* is the polymer mass concentration, and *K* is the optical constant. Refractive index increments *∂n/∂c* necessary to calculate *K* were determined using Schulz-Cantow differential refractometer at different *w*_H_2_O_ at 25 °C. The results are listed in the second line of Table 1. In general, *∂n/∂c* for perfluorinated polymers in water or alcohol is so small that SLS measurements had to be made at relatively high concentrations.

It is known that *M*_w_ values obtained by SLS for copolymers in mixed solvents are affected by both the selective solvation effect and the composition dispersity effect [14]. However, water and MeOH have similar refractive index increments, and the degree of substitution of the sulfonated side group is so low that we have neglected the above two effects on *M*_w_ obtained from SLS.

The intensity auto-correlation function *g*^(2)^(*t*) obtained by DLS was analyzed by the CONTIN program to determine the spectrum *A*(*R*_H,app_) of the apparent hydrodynamic radius *R*_H,app_ defined by [15].

(2) $$R_{H,\mathrm{app}}\equiv \frac{k_{B}T}{6\pi \eta_{S}}k^{2}\tau ,\;\ln{\left[\frac{g^{\left(2\right)}(t)-1}{g^{\left(2\right)}(0)-1}\right]}^{1/2}=\sum_{R_{H,\mathrm{app}}}A\left(R_{H,\mathrm{app}}\right)\exp\left(-\frac{k_{B}T}{6\pi \eta_{S}}\frac{k^{2}t}{R_{H,\mathrm{app}}}\right)$$

where *t* is the delay time, *τ* is the relaxation time, *k* is the magnitude of the scattering vector, *k*_B_*T* is the Boltzmann constant multiplied by the absolute temperature, and η_S_ is the solvent viscosity. The true hydrodynamic radius can be obtained only when *R*_H,app_ is extrapolated to the zero polymer concentration and zero *k*, but such extrapolation was not made in this study because of the very strong inter-particle electrostatic interaction.

### 2.3. SAXS Measurements

SAXS measurements were conducted on PFSI solutions at the BL40B2 beamline of SPring-8 (Hyogo, Japan) with the approval of JASRI (Proposal Nos. 2015B1100 and 2015B1674). The polymer concentration was fixed to be 0.0050 g/cm^3^. The wavelength of the X-ray, the camera length, and the accumulation time were set to be 0.1 nm, 4000 mm, and 180 s, respectively. A capillary made of quartz that contained test solutions was set in a heating block at 25 °C, and the intensity of the scattered X-ray was measured using an imaging plate detector.

The excess Rayleigh ratio *R*_X,θ_ at the scattering angle θ and the optical constant *K*_e_ of SAXS were calculated by

(3) $$R_{X,\theta }=F\left(\frac{I_{\theta ,\mathrm{soln}}}{I_{\mathrm{mon},\mathrm{soln}}}-\frac{I_{\theta ,\mathrm{solv}}}{I_{\mathrm{mon},\mathrm{solv}}}\right),\;K_{e}=N_{A}{a_{e}}^{2}\gamma^{2}$$

Here, *F* is the instrument constant (which was determined by the comparison with the SLS results; see below), *I*_θsoln_ (*I*_θ,solv_) and *I*_mon,soln_ (*I*_mon,solv_) are the scattering intensity at the scattering angle θ and the monitor value of the incident SAXS intensity, respectively, of the solution (of the solvent), *N*_A_ is the Avogadro constant, *a*_e_ is the classical radius of electron, and *γ* is the SAXS contrast factor of the polymer. Values of *γ* for PFSI in water–MeOH mixtures with *w*_H_2_O_ were calculated by [16]

(4) $$\gamma =\frac{\left(1-x\right)n_{e,\mathrm{TEF}}+xn_{e,S}}{\left(1-x\right)M_{\mathrm{TFE}}+xM_{S}}-\frac{\bar{\upsilon }}{\upsilon_{\mathrm{solv}}}\left[\frac{n_{{e,H}_{2}O}}{M_{H_{2}O}}w_{H_{2}O}+\frac{n_{e,\mathrm{MeOH}}}{M_{\mathrm{MeOH}}}\left(1-w_{H_{2}O}\right)\right]$$

where the subscripts TFE and S denote the monomer units of tetrafluoroethylene and sulfonated-side-chain substituted TFE, respectively, *x* is the mole fraction of S in the copolymer, *n*_e,*i*_ and *M_i_* (*i* = TFE, S, H_2_O, and MeOH) are numbers of electrons and molar masses of *i*, respectively, $\bar{\upsilon }$ is the partial specific volume of PFSI, and *ν*_solv_ is the specific volume of the solvent. The value of $\bar{\upsilon }$ was determined in water by densitometry and assumed to be independent of *w*_H_2_O_. Again, the selective solvation effect was neglected. Values of *γ* such calculated are listed in the third line of Table 1. In contrast to the SLS contrast factor *∂n/∂c*, *γ* of PFSI in water–MeOH mixtures takes normal values comparable to usual synthetic polymers.

## 3. Results and Discussion

### 3.1. SLS

**Figure 1:**
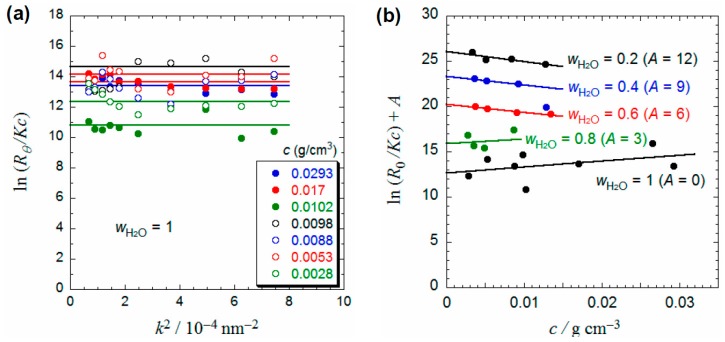
(**a**) Guinier plots of PFSI in water at different polymer concentrations *c* at 25 °C; and (**b**) plots of ln(*R*_0_*/Kc*) vs. *c* of PFSI in water–MeOH mixtures with different *w*_H_2___O_ at 25 °C.

Figure 1a shows the Guinier plots of PFSI in water at different polymer mass concentrations *c* at 25 °C. Here, *R*_θ_ is the excess Rayleigh ratio, *K* is the optical constant, and *k* is the magnitude of the scattering vector. Because of weak scattering power of PFSI, data points scatter more or less, but we can say that *R*_θ_ is almost independent of *k* or the scattering angle θ. This indicates that the particle size of PFSI in water is not so large. Similar angular independent Guinier plots were obtained for PFSI solutions with *w*_H_2_O_ = 0.2–0.8.

Because of no appreciable angular dependence, we have estimated ln(*R*_0_/*Kc*) in Equation (1) by averaging ln(*R*_θ_/*Kc*) at different scattering angle θ. Figure 1b shows the concentration dependence of ln(*R*_0_/*Kc*) such obtained at different *w*_H_2_O_. At *w*_H_2_O_ = 0.2, 0.4, and 0.6, ln(*R*_0_/*Kc*) slightly decreases with *c*, indicating that the second virial coefficient *A*_2_ is positive from Equation (1). Although data points are rather scattered at *w*_H_2___O_ = 0.8 and 1, they seem to follow straight lines with smaller or even negative slopes.

**Figure 2:**
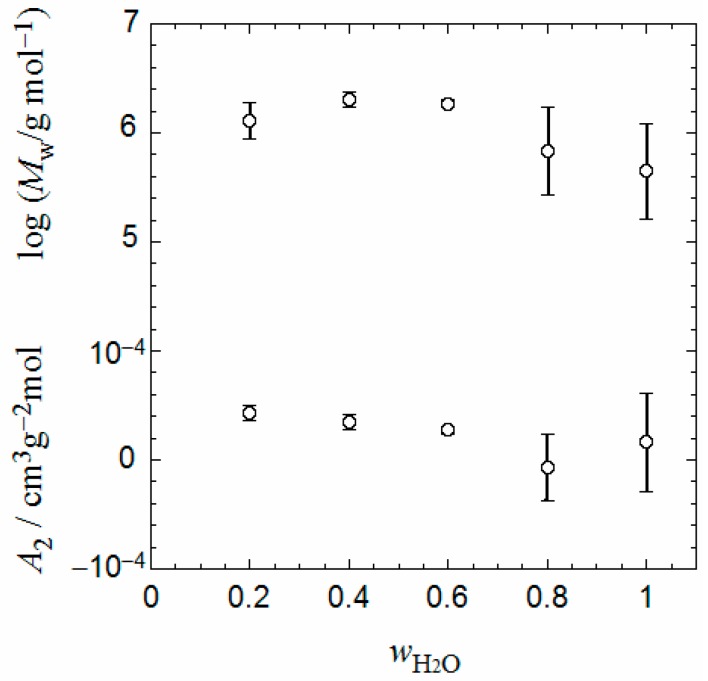
Solvent composition dependences of the weight average molar mass *M*_w_ and second virial coefficient *A*_2_ of PFSI at 25 °C.

Using Equation (1), the weight average molar mass *M*_w_ and *A*_2_ were obtained from the plots of ln(*R*_0_/*Kc*) vs. *c* given in Figure 1b, and their solvent composition dependences are given in Figure 2. Both *M*_w_ and *A*_2_ are almost independent of *w*_H_2___O_ at 0.2 ≤ *w*_H_2___O_ ≤ 0.6, and slightly decrease with further increasing *w*_H_2___O_, though errors are considerably large. The weak solvent composition dependences imply that solvent quality is not so much different between water and MeOH for this fluoronated ionomer. Because the average molar mass per the PFSI monomer unit is 146 g/mol, the PFSI particle existing in water–MeOH mixtures with 0.2 ≤ *w*_H_2___O_ ≤ 1 consists of ca. 3000–9000 monomer units, although its aggregation number cannot be estimated because we do not know the molecular weight of this PFSI sample. The values of *M*_w_ are comparable to that for a heat-treated PFSI dispersion reported by Curtin et al. [12].

The values of *A*_2_ are comparable to those for usual non-ionic polymers in good or poor solvents, which indicate that the strong electrostatic and hydrophobic interactions of PFSI particles in water and water–MeOH mixtures may cancel each other. The theory of Derjaguin, Landau, Verwey, and Overbeek for the stability of spherical colloids [17] demonstrates that the van der Waals attraction is long-ranged compared to the electrostatic repulsion when two spherical particles approach closely.

Curtin et al. [12] reported that the radius of gyration of a PFSI dispersion with a broad molar mass distribution before heat treatment is proportional to the molar mass, indicating that the PFSI dispersion exists as rod-like aggregates in dimethylformamide (DMF). However, they did not mention the molar mass dependence of the radius of gyration after heat treatment, and our PFSI dispersion has a molar mass similar to that of Curtin et al. after heat treatment. Although Curtin et al. did not show the radius of gyration data nor the angular dependence of SLS, they used incident light of the wavelength 800 nm, so that the radius of gyration estimated from SLS must be much larger than 10 nm. The weak angular dependence in Figure 1a indicates that our PFSI dispersion does not include such rod-like aggregates of Curtin et al. before heat treatment.

### 3.2. DLS

**Figure 3:**
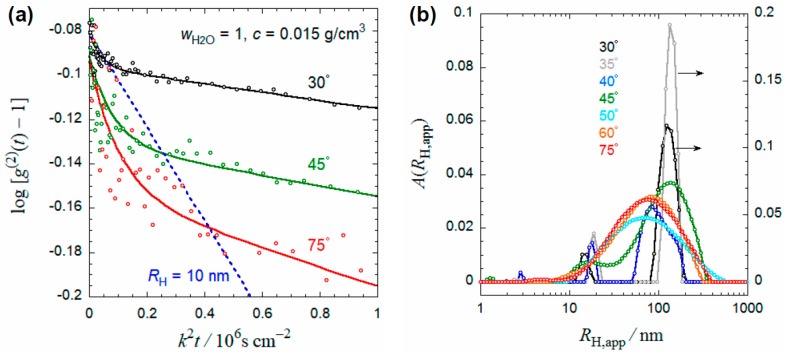
Dynamic light scattering (DLS) results for a PFSI solution with *w*_H_2___O_ = 1 and *c* = 0.015 g/cm^3^.

Intensity auto-correlation functions *g*^(2)^(*t*) and spectra of the apparent hydrodynamic radius *A*(*R*_H,app_) for a PFSI solution with *w*_H_2___O_ = 1 and *c* = 0.015 g/cm^3^ are shown in Figure 3. Due to the small value of *∂n/∂c* (see Table 1), scattering intensities were so weak that correlation functions contain considerable experimental errors especially at high θ, but they definitely consist of multiple relaxation modes. According to the CONTIN analysis, *A*(*R*_H,app_) has two peaks at θ ≤ 45°, and the two peaks merge at θ > 45° to give a broad distribution ranging from 10 nm to few hundred nm.

The peak of *A*(*R*_H,app_) around *R*_H,app_ = 100 nm seems to be inconsistent with the weak angular dependence of ln(*R*_θ_/*Kc*) shown in Figure 1a. Long years ago, Pusey [18] reported similar *g*^(2)^(*t*) for a charged polystyrene latex in water (*c* = 1.25 × 10^−3^ g/cm^3^; see Figure 2 of Reference [18]). The decay of the free diffusion corresponding to the true hydrodynamic radius was close to the short-time decay of *g*^(2)^(*t*), and the log-time decay was ascribed to the hopping of the particle temporarily trapped in a repulsive cage formed by its neighbors. Because the PFSI particle also has electric charges, the same “repulsive cage effect” may be expected. In fact, the short-time decay in *g*^(2)^(*t*) shown in Figure 3a has a similar slope to the dotted blue line, indicating the decay corresponding to the free diffusion of a particle with *R*_H_ = 10 nm, being consistent with the SLS result. Previous DLS studies on aqueous PFSI solutions often reported the existence of large particles in the solutions, but it may be questionable because of the “repulsive cage effect.”

### 3.3. SAXS

**Figure 4:**
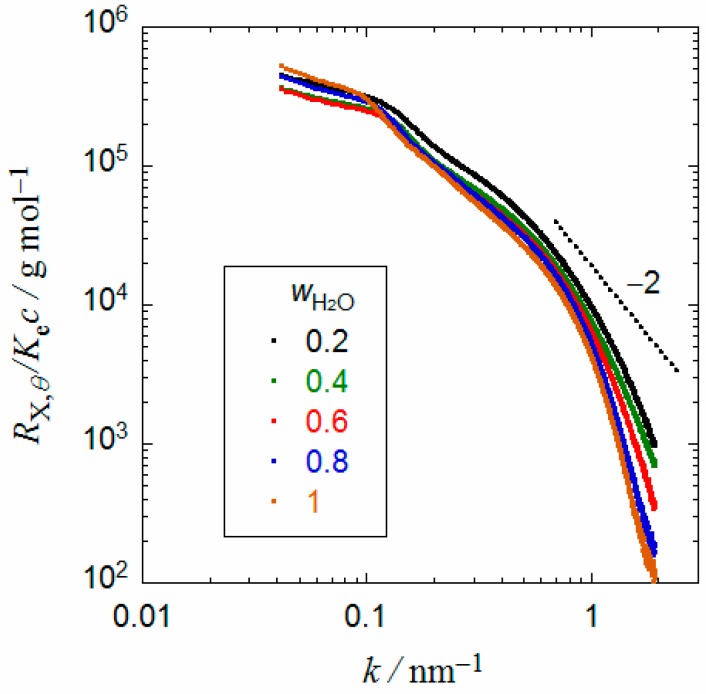
SAXS profiles of PFSI in water and water–MeOH mixtures at *c* = 0.005 g/cm^3^ at 25 °C.

Figure 4 shows SAXS scattering functions for PFSI in water and water–MeOH mixtures at *c* = 0.005 g/cm^3^. (The instrument constant *F* in Equation (3) was determined to agree the average intercept of *R*_X,θ_/*K*_e_c with that of the SLS *R*_θ_/*K*c shown in Figure 1a.) Although the scattering functions are not so much dependent of the solvent composition, their decays in high *k* region are steeper at higher *w*_H_2___O_, indicating the larger particle size at the higher *w*_H_2___O_. Because slopes of all the scattering functions in high *k* region are steeper than −2 (the dashed line in the Figure), PFSI does not exist as a random coil but takes a more compact conformation in water and water–MeOH mixtures. All the scattering functions seem to consist of two decaying curves at *k* smaller and larger than 0.2 nm^−1^ and possess weak peaks around *k* = 0.12 nm^−1^. The peaks may arise from the long range electrostatic interaction among PFSI particles. Similar peaks in SAXS profiles for PFSI in water and water–alcohol mixtures were reported in previous literature [3,4,7,8].

As already mentioned in Introduction, the previously reported rodlike model for the PFSI particle is questionable. Furthermore, SLS and DLS results indicated that the PFSI particle size may not be so large. Thus, it may be more natural to assume the PFSI particle to be spherical with lower interfacial energy. Because the scattering functions seem to consist of two decaying curves, the molar mass distribution of the spherical particles is assumed to be bimodal. Then, the particle scattering function *P*(*k*) is given by

(5) $$M_{w}P(k)=\left(1-w_{\mathrm{large}}\right)M_{w,\mathrm{small}}P_{z,\mathrm{small}}(k)+w_{\mathrm{large}}M_{w,\mathrm{large}}P_{z,\mathrm{large}}(k)$$

where *w*_large_ is the weight fraction of the large spherical component in the total spherical components, *M*_w,*i*_ and *P_z_*_,i_(*k*) (*i* = small and large) are the weight average molar mass and z-average particle scattering function of the spherical component *i*, respectively. The particle scattering function *P*_z,i_(*k*) may be written as [16,19,20,21]

(6) $$P_{z,i}(k)=\int 9{\left[\frac{\sin(kR_{M})-kR_{M}\cos(kR_{M})}{{\left(kR_{M}\right)}^{3}}\right]}^{2}Mw_{i}(M)dM$$

where *R*_M_ is the radius of the fraction with the molar mass *M*, which is calculated by

(7) $$R_{M}={\left(\frac{3M}{4\pi N_{A}c_{\mathrm{in}}}\right)}^{1/3}$$

with the Avogadro constant *N*_A_ and the polymer mass concentration *c*_in_ inside the spherical particle, being assumed to be common for both components, and *w*_i_(*M*) is the molar mass distribution function (the weight fraction of the fraction with molar mass *M* in the component *i*). Assuming the log-normal distribution for w_i_(*M*), we write

(8) $$Mw_{i}(M)={\left[2\pi \ln\left(\frac{M_{w,i}}{M_{n,i}}\right)\right]}^{-1/2}\exp\left[-\frac{\ln^{2}\left(M/{M_{i}}^{\circ }\right)}{2\ln\left(M_{w,i}/M_{n,i}\right)}\right],{M_{i}}^{\circ }\equiv \sqrt{M_{w,i}M_{n,i}}$$

with *M*_w,*i*_ and *M*_n,i_ being the weight-average and number-average molar masses of the spherical component *i*. Using the Zimm approximation for the inter-particle interference factor [22], we have

(9) $$\frac{R_{X,\theta }}{K_{e}c}=\frac{M_{w}P(k)}{1+2A_{2}M_{w}P(k)c}$$

**Figure 5:**
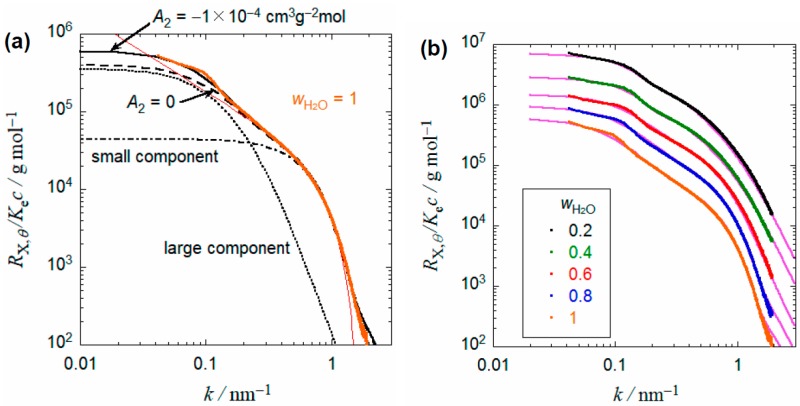
Fitting results for SAXS scattering functions.

The fitting result for the scattering function in water is shown in Figure 5a. The above Equations (5)–(9) have 7 adjustable parameters, *w*_large_, *c*_in_, *M*_w,small_, *M*_n,small_, *M*_w,large_, *M*_n,large_, and *A*_2_. As shown by the dot-dash and dotted curves, the scattering function in the high and low *k* regions are determined mainly by the small and large components characterized by *M*_w,i_ and *M*_n,i_. The decay and relative height of the dot-dash and dotted curves are governed by *c*_in_ and *w*_large_. The scattering function in the low *k* region is also dependent on *A*_2_, but the *A*_2_ value was chosen to be close to that estimated by SLS. The solid curve cannot reproduce the small peak of the scattering function around *k* = 0.12 nm^−1^. This is due to the Zimm approximation used. The ambiguity in the inter-particle interference factor may introduce some errors in the parameters of the large component, but not those of the small component.

Loppinet et al. [8] fitted their SANS scattering function in a high *k* region by the particle scattering function for the cylindrical particle given by

(10) $$P(k)\propto \frac{1}{kr}{\left[\frac{J_{1}(kr)}{kr}\right]}^{2}$$

where *r* is the cylinder radius and *J*_1_(*x*) is the first-order Bessel function. As shown by the red thin curve in Figure 5a, our SAXS scattering function can be fitted by this function with *r* = 2.3 nm in a limited range of *k* similar to that examined by Loppinet et al. Because our fitting by the spherical model is even better, Loppinet et al.’s fitting does not verify the rodlike aggregate model. Rebrov et al. [4] compared their SAXS profile for a PFSI dispersion in DMF with theoretical curves of a thin disc and flat prism. The agreements between the experiment and theory were much less satisfactory.

**Figure 6:**
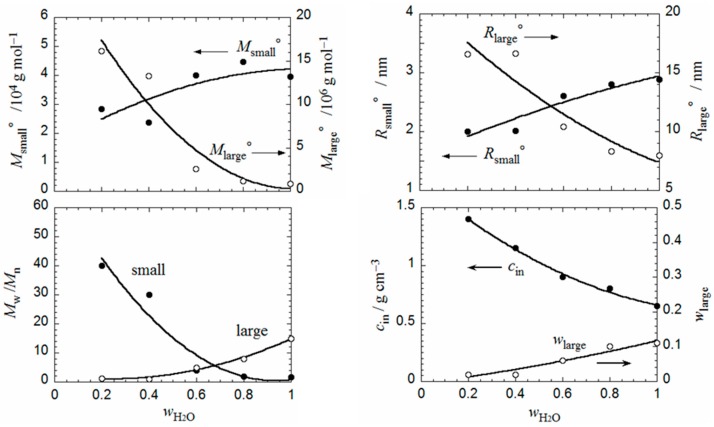
Solvent composition dependences of parameters for PFSI spherical particles determined by fitting of SAXS profiles shown in Figure 5b.

Figure 6 shows the solvent composition dependences of parameters for PFSI spherical particles determined by fitting of SAXS profiles shown in Figure 5b, as well as of the average radii calculated by

(11) $${R_{i}}^{\circ }={\left(\frac{3{M_{i}}^{\circ }}{4\pi N_{A}c_{\mathrm{in}}}\right)}^{1/3}$$

For the large component, *M*_large_^°^ and *R*_large_^°^ increase and *M*_w,large_*/M*_n,large_ and *w*_large_ decrease with increasing the MeOH content, while for the small component, the corresponding quantities change oppositely. Furthermore, *c*_in_ increases with increasing the MeOH content. Since the dry density of PFSI is as high as 2 g/cm^3^, the PFSI particle more or less swells in water and water–MeOH mixtures, maybe due to sulfonate groups in the PFSI particle. Both *R*_small_^°^ and *R*_large_^°^ are smaller than 17 nm, which are consistent with the weak angular dependences in the Guinier plots of SLS shown in Figure 1a.

**Figure 7:**
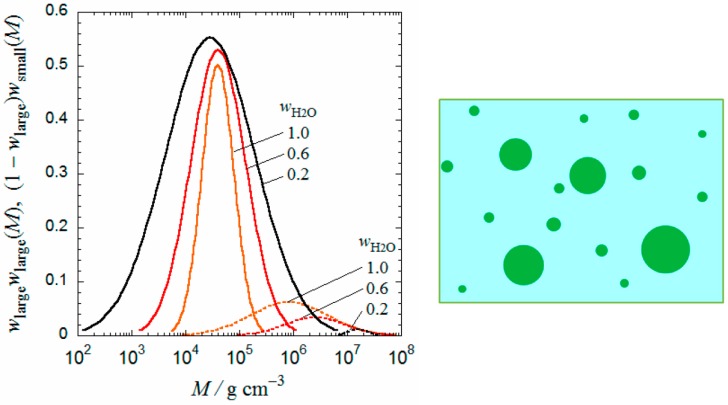
Weight fractions (1 − *w*_large_)*w*_small_(*M*) (solid curves) and *w*_large_*w*_large_(*M*) (dotted curves) of the fraction with *M* for the small and large components, respectively, calculated parameters shown in Figure 6. Right Panel, schematic diagram of the bidisperse spherical particles model.

Figure 7 shows the weight fractions (1 − *w*_large_)*w*_small_(*M*) and *w*_large_*w*_large_(*M*) of the fraction with *M* for the small and large components calculated parameters shown in Figure 6. The higher molar mass peak of the large component diminishes from the low *M* side by addition of MeOH, while the lower molar mass peak of the small component becomes broader with decreasing *w*_H_2___O_, because both of the dissociation of the small component and of the incorporation of the dissociated fractions of the large component. We may say that the addition of MeOH into the aqueous dispersion of PFSI slightly weakens the hydrophobic interaction of PFSI to dissociate PFSI spherical particles. Kyriakos et al. [23] reported that the micelle of polystyrene-*b*-poly(*N*-isopropylacrylamide) formed in water aggregates by addition of MeOH and ethanol and ascribed the aggregation to the dehydration from the coronal chains of the micelle by the alcohols added. The dissociation of both small and large components from the low *M* side in our system implies that the hydration force of PFSI does not play a role of the colloidal stabilization.

With increasing the MeOH content, the dielectric constant of the water–MeOH mixture decreases, which reduces the electrostatic interaction of PFSI. This reduction may reflect the increase in *c*_in_. MeOH may weaken both the hydrophobic and electrostatic interactions of PFSI in the aqueous dispersion, and it is rather difficult to classify whether MeOH is a good or poor solvent (dispersant) for PFSI.

## Figures and Tables

**Table 1:** Refractive index increments *∂n/∂c* and small-angle X-ray scattering (SAXS) contrast factors *γ* of PFSI in water–methanol (MeOH) mixtures with different *w*_H_2___O_ at 25 °C.

*w*_H_2___O_	0.2	0.4	0.6	0.8	1
*∂n/∂c* ^a^	0.0110	0.0085	0.0087	0.0110	0.0136
*γ* ^b^	0.145	0.163_5_	0.178	0.189	0.198

^a^ Value at 532 nm wavelength, in units of cm^3^/g; ^b^ SAXS contrast factor calculated by Equation (4), in units of mol/g.

**Figure A1:**
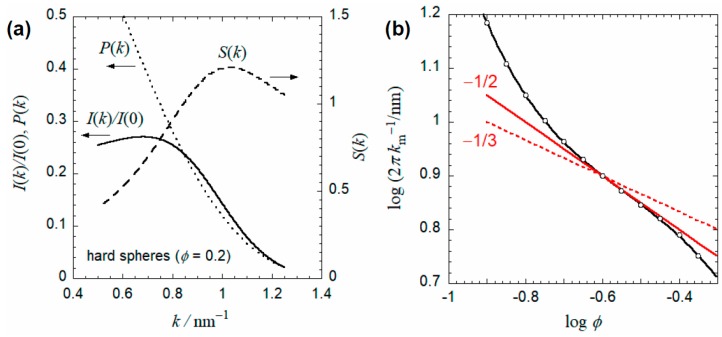
Double logarithmic plot of 2π*/k*_m_ vs. ϕ for hard sphere solutions.

## Appendix A

The normalized scattering function *I*_θ_*/I*_0_ for colloidal solutions is written as

(A1) $$I_{\theta }/I_{0}=S(k)P(k)$$

where *S*(*k*) is the structure factor and *P*(*k*) is the particle scattering function. For the solution of hard spheres with the radius *R*, *S*(*k*) and *P*(*k*) are given by [24]

(A2) $$\begin{array}{c} \frac{1}{S(k)}=1+\frac{24\phi {\left(1+2\phi \right)}^{2}}{{\left(1-\phi \right)}^{4}}\frac{\sin q-q\cos q}{q^{3}}-\frac{144\phi^{2}{\left(1+\frac{1}{2}\phi \right)}^{2}}{{\left(1-\phi \right)}^{4}}\frac{2q\sin q-(q^{2}-2)\cos q-2}{q^{4}}\\ +\frac{6\phi^{2}{\left(1+2\phi \right)}^{2}}{{\left(1-\phi \right)}^{4}}\frac{(4q^{3}-24q)\sin q-(q^{4}-12q^{2}+24)\cos q+24}{q^{6}} \end{array}$$

(A3) $$P(k)=\frac{9}{{\left(kR\right)}^{6}}{\left(\sin kR-kR\cos kR\right)}^{2}$$

with the volume fraction ϕ of the spheres in the solution and *q* ≡ 2*kR*.

The structure factor *S*(*k*) given by Equation (A2) has a peak, and the peak position is proportional to ϕ^1/3^ in a high ϕ region. The scattering function given by the above equations has also a peak, but the concentration dependence of the peak position *k*_m_ does not obey the ϕ^1/3^ dependence. Figure A1 shows the scattering function at ϕ = 0.2 and the concentration dependence of 2π*/k*_m_ obtained from the scattering function calculated by Equations (A1)–(A3). The double logarithmic plot of 2π*/k*_m_ vs. ϕ is not linear, and has a slope of ca. −1/2 in an intermediate concentration range. There is no concentration range where the slope of the plot is −1/3, which Aldebert et al. [3] expected for spherical particle solutions.

Hayter and Penfold formulated the structure factor for the charged spherical particle solution [25]. Using their result instead of Equation (A2), we obtained similar *I*_θ_*/I*_0_ to lead the same conclusion on the concentration dependence of the peak position also for the charged spherical particle system.
